# Are Gettier cases disturbing?

**DOI:** 10.1007/s11098-020-01493-0

**Published:** 2020-06-26

**Authors:** Peter Hawke, Tom Schoonen

**Affiliations:** 1 Institute for Logic, Language, and Computation, University of Amsterdam, Amsterdam, The Netherlands; 2 Arché, University of St. Andrews, St. Andrews, UK

**Keywords:** Gettier cases, Experimental philosophy, Naturalism, Thought experiments, Philosophical methodology, Methods of cases, Moderate modal skepticism

## Abstract

We examine a prominent naturalistic line on the method of cases (MoC), exemplified by Timothy Williamson and Edouard Machery: MoC is given a fallibilist and non-exceptionalist treatment, accommodating moderate modal skepticism. But Gettier cases are in dispute: Williamson takes them to induce substantive philosophical knowledge; Machery claims that the ambitious use of MoC should be abandoned entirely. We defend an intermediate position. We offer an internal critique of Macherian pessimism about Gettier cases. Most crucially, we argue that Gettier cases needn’t exhibit ‘disturbing characteristics’ that Machery posits to explain why philosophical cases induce dubious judgments. It follows, we show, that Machery’s central argument for the effective abandonment of MoC is undermined. Nevertheless, we engineer a restricted variant of the argument—in harmony with Williamsonian ideology–that survives our critique, potentially limiting philosophy’s scope for establishing especially ambitious modal theses, despite traditional MoC’s utility being partially preserved.

## Introduction

*Naturalism* is the vague but suggestive doctrine that philosophy ought to be continuous with science. It is best identified with a loose cluster of typical impulses (Rysiew 2017), including hostility to accounts of philosophical methodology that posit faculties or methods that are unanswerable to (or take priority over) the best scientific theories and methods. Naturalists are thus typically suspicious of the rationalistic claim that philosophical knowledge emanates from an infallible kind of a priori insight, intuition, or reflection that philosophers are specially attuned to.^1^

Two naturalistic lines of research on the method of cases (MoC) are currently prominent. Theorists on the **F-line** focus on the *form* and *content* of (the reasoning induced by) philosophical thought experiments. For example, they aim to provide a rational reconstruction of the ‘Gettier-reasoning’ that supports the standard ‘Gettier judgment’ (knowledge is not justified true belief) in response to Gettier cases (Gettier 1963). Williamson (2007) and Geddes (2017) develop a broadly naturalistic version of the F-line, eschewing appeal to ‘rational insight’ based on, for instance, a *sui generis* faculty of intuition (Bealer 1998; BonJour 1998; Bealer 2002) or mere conceptual competence (Boghossian 1996).^2^ Instead, their accounts are *non-exceptionalist*: they only appeal to ordinary cognitive capacities whose nature and reliability is amenable to scientific (e.g. evolutionary) explanation.^3^ Meanwhile, theorists on the **X-line** (e.g. Weinberg et al. 2001; Swain et al. 2008; Wright 2010; Starmans and Friedman 2012; Nagel et al. 2013; Turri 2013; Machery 2017) focus on the question of *robustness*: which philosophical thought experiments, if any, elicit judgments that are stable and uniform across population and presentation? Standard scientific tools (i.e. rigorous experimental design and analysis) are deployed to clarify and assess MoC’s trustworthiness, again on the naturalistic assumption that MoC utilizes *ordinary* judgment (lest folk surveys be rendered irrelevant).

F-liners and X-liners proceed from opposing inclinations. F-liners typically assume that prominent instances of MoC induce good reasoning, yielding knowledge in paradigm cases. Recovering this possibility is taken as a mark of an adequate reconstruction. X-liners assume a sceptical stance: it is a matter of (empirical) scrutiny whether MoC deserves its cherished status in the philosopher’s tool kit.

Our broad aim is to clarify the interaction between the F-line and X-line, and gesture at the common path forward for naturalists. Our narrow aim is to explore, in particular, how F-liner Williamson (2007) and X-liner Machery (2017) complement and contrast with each other: we identify crucial shared commitments; rule on a disagreement about the force of the Gettier thought experiment (henceforth: Gettier); and thereby examine how far Williamsonians should accept radical Macherian conclusions. Williamson and Machery are fruitful stalking horses. Their common ground offers an attractive foundation for a moderate naturalism about MoC, the implications of which deserves close scrutiny.^4^ Further, Williamson’s account has been especially influential. Despite criticism of its details (Jenkins 2008; Ichikawa and Jarvis 2009; Malmgren 2011; Roca-Royes 2011; Vetter 2017), it remains a basic model for refinement for naturalists. Meanwhile, Machery’s *Philosophy Within Its Proper Bounds* (henceforth *PwPB*) is a milestone for experimental philosophy, basing radical methodological conclusions on nuanced argumentation and a comprehensive overview of existing experimental results, including large-scale studies reported by Machery et al. (2017, 2018a, b). In particular, Machery (2017, pp. 6–8) advocates *radical restrictionism*: in light of its empirically confirmed unreliability, traditional MoC should effectively be shelved, and judgment about standard philosophical cases suspended, Gettier cases included.^5^ In contrast, some prominent X-liners only endorse *moderate restrictionism* (Weinberg 2007; Alexander and Weinberg 2014): existing empirical results don’t establish the widespread unreliability of philosophical thought experiments, but show that identifying trustworthy instances is a non-trivial empirical task.

Section 2 isolates common ground between the Williamsonian F-line and the Macherian X-line. Section 3 uses it to explicate and criticize a Macherian case for pessimism about Gettier. Section 3.1 argues that Macherian pessimism hinges on the claim that Gettier cases have intrinsic features that disturb ordinarily reliable judgment. Section 3.2 argues that key Gettier cases are *not* disturbing. Section 4 considers implications for central arguments in *PwPB*. Section 4.1 argues that Machery’s argument for radical restrictionism is undermined if Gettier can paradigmatically be taken as reliable. However, Sect. 4.2 presents a cautious variant of Machery’s argument, in support of a potent modal ignorance that limits philosophy’s theoretical ambitions, despite some preservation of traditional MoC. On a Williamsonian model, the resulting moderate modal skepticism subtly contrasts with a more familiar form advocated by van Inwagen (1998) and Hawke (2011, 2017).

## Common ground

On a broadly Williamsonian approach, a successful account of MoC has three features.^6^ First, it is non-exceptionalist. Second, it paints MoC as delivering (what Machery calls) *material-mode* conclusions: MoC, it is held, is not used “to discover the meaning of words or the semantic content of concepts of philosophical interest, but to understand their referents” (Machery 2017, p. 16). Relatedly, applications of MoC are taken to establish metaphysical possibilities, the “sort of possibility most relevant to the nature of the phenomena under investigation” (Williamson 2007, p. 206). Third, the account must explain the paradigmatic success of Gettier, on the hypothesis that “if any thought experiment can succeed in philosophy, then [Gettier’s] do” (Williamson 2007, p. 178).

Points of affinity with Machery (2017) are immediate. Machery agrees that MoC is best described as non-exceptionalist: the induced judgments “are warranted, if they are, for the very reason that everyday judgments are warranted, whatever that is” (Machery 2017, p. 21). He agrees that MoC is best characterized as in the material-mode (2017, p. 16). Though cautious in his conclusions, he agrees that Gettier stands out as particularly robust: the judgments elicited by Gettier cases have only negligible demographic variation (Machery et al. 2018a) and only small to moderate ordering and framing effects (see Table 2.9 on pp. 86–87 of Machery 2017). The folk apparently judge in accord with philosophical orthodoxy at a similar rate to their judgment of ignorance in response to a trivial ‘false belief’ case. This contrasts with early experimental studies that concluded significant demographic variation in judgment (Weinberg et al. 2001), but used small sample sizes and failed to be replicated (Nagel et al. 2013; Turri 2013; Kim and Yuan 2015; Sayadsayamdost 2015). Indeed, Machery et al. (2018a) hypothesize that the Gettier judgment reflects universal features of folk epistemology (Machery et al. 2017 are more cautious).

To elaborate, consider a key Gettier case:

**Hospital.** Paul Jones was worried because it was 10 pm and his wife Mary was not home from work yet. Usually she is home by 6 pm. He tried her cell phone but just kept getting her voicemail. Starting to worry that something might have happened to her, he decided to call some local hospitals to ask whether any patient by the name of “Mary Jones” had been admitted that evening. At the University Hospital, the person who answered his call confirmed that someone by that name had been admitted with major but not life-threatening injuries following a car crash. Paul grabbed his coat and rushed out to drive to University Hospital. As it turned out, the patient at University Hospital was not Paul’s wife, but another woman with the same name. In fact, Paul’s wife had a heart attack as she was leaving work, and was actually receiving treatment in Metropolitan Hospital, a few miles away.

Philosophical orthodoxy takes Hospital to induce the judgment that Paul has a justified true belief (his wife is in hospital) that isn’t knowledge. Call this a *singular Gettier judgment*, supporting the *universal Gettier judgment*: knowledge is not justified true belief. As it is tricky to explain precisely why Paul lacks knowledge (Shope 1983), suggestive but non-committal terminology will be useful: Paul’s belief is not knowledge since its grounds are not suitably *sensitive* to what makes it true—its truth is somehow *lucky*.

Credible studies indicate that Hospital induces widespread convergence on the singular Gettier judgment, bolstering philosophical orthodoxy.^7^ Surveying over 2000 participants, Machery et al. (2017) find both men and women made the singular Gettier judgment at a rate of about 80%. Participants across 23 countries and 16 languages made the singular Gettier judgment at rates between 70% and 90%.^8^ Machery et al. (2018a) report similar cross-cultural invariance: 86% of US respondents issued the singular Gettier judgment; 95% of Brazilians; 88% of Indians; 91% of Japanese.

Hospital represents an important class of Gettier case. In the terminology of Turri (2019), it exhibits the structure: *no detect with replacement*.^9^ Though the agent is reasonable to believe the proposition in question, they fail to genuinely detect its truth. The *presumed* truthmaker for the proposition has not in fact been realized; it is true in virtue of a ‘replacement’ truthmaker. Paul justifiably believes his wife is hospitalized, on the basis of a reasonable presumption that she was admitted to University. His presumption is incorrect: she was admitted to Metropolitan. This class is doubly notable. First, it plausibly includes the original counter-examples of Gettier (1963). Hence, the philosophical work achieved by Gettier’s paper is equally achieved by the robust inducement of a singular Gettier judgment by Hospital. Second, there is evidence that cases in this class tend to induce the singular Gettier judgment with striking frequency: see Starmans and Friedman (2012), Turri (2013), Turri et al. (2015) for a selection.^10^ This contrasts, Turri et al. (2015) show, with Gettier cases with so-called *detection with failed threat* structure (e.g. the fake-barn cases of Goldman (1976)) or *detection with replacement* structure (e.g. the ‘authentic evidence’ cases of Starmans and Friedman (2012)). Turri (2019) rightly cautions: that a certain type of Gettier case induces (or fails to induce) largely uniform judgment doesn’t support conclusions about the abstract class of Gettier cases as a whole—in particular, those with very different epistemic structure. We nowhere assume that conclusions about Hospital translate into clear morals for, say, fake-barn cases (or vice versa).

Strikingly uniform folk judgment about Hospital doesn’t indicate *accurate* judgment if folk epistemic judgment is systematically inaccurate. However, Williamson and Machery accept (what Alexander and Weinberg (2014) call) the *general reliability thesis*: blind-spots granted, folk epistemic judgment is generally accurate when evaluating suitably mundane cases.^11^ Crucially, *non-exceptionalism* and the *general reliability thesis* yield:

*Epistemic non-exceptionalism.* Absent specific defeat, a MoC judgment about a mundane case is rightly treated as expert judgment.

Epistemic non-exceptionalism would be questionable if promising accounts of MoC that entail it were elusive. Fortunately, Williamson (2007, Ch. 6) offers such an account. The reasoning induced by Hospital is explicated roughly as:

**W1**: Hospital is (metaphysically) possible.
**W2**: If Hospital were the case, then someone would justifiably believe a true proposition without knowing it.
**C1**: Thus: it is (metaphysically) possible for someone to justifiably believe a true proposition without knowing it.
**C2**: Thus: it is not (metaphysically) necessary that one knows *p* just in case *p* is true and one justifiably believes *p*.

Generally, Gettier-reasoning proceeds as follows: the subject judges both that the described case is possible ($\mathbf{W1}$) and that if it were to occur, then someone would have a justified belief in true proposition *p* without knowledge of *p* ($\mathbf{W2}$). The subject thereby draws a singular Gettier judgment ($\mathbf{C1}$). The universal Gettier judgment follows ($\mathbf{C2}$).

**W1** is justified by whatever justifies ordinary objective possibility claims (perhaps: reality-oriented imagination or ampliative reasoning). Williamson proposes that **W2** is justified via an exercise of reality-oriented imagination, guiding a simulated rational belief update: “one supposes the antecedent and develops the supposition, adding further judgments within the supposition by reasoning, offline predictive mechanisms, and other offline judgments” (2007, pp. 152-153). What grounds the accuracy of such simulations? For Gettier, we can partly appeal to our ordinary capacity for mindreading (Nagel 2012). Indeed, given an *actual* Gettier case, the modal and counterfactual aspects of the reasoning are trivialized, with **W2**’s justification plausibly collapsing into mere mindreading.

Williamson’s account has met resistance. We needn’t be distracted. First, it is ‘proof-of-concept’ for the Williamsonian approach, whatever refinements await. Second, the objections chiefly target the appeal to counterfactual reasoning, but such worries can be postponed by focusing on actualized Gettier cases. Third, the chief criticisms may not necessitate radical refinement. To illustrate, the account has been criticized for erroneously predicting that *deviant realizations* can defeat Gettier-reasoning (Ichikawa and Jarvis 2009; Malmgren 2011). A deviant realization of Hospital satisfies its bare description but includes details that necessitate that the agent does *not* have a justified true belief without knowledge (e.g., Paul knows by an *unmentioned* source that his wife is in hospital). Now suppose that (only) deviant realizations are actual. Thus **W2** is false, and the Williamsonian must conclude that the Gettier-reasoning fails. This is counter-intuitive: if deviant realizations are actualized, this seems *irrelevant* to Gettier’s force. Here are three strategies for amending Williamson’s analysis. The first targets the appeal to a counterfactual conditional, perhaps deploying a more subtle conditional (cf. Geddes 2017). The second amends the content of the counterfactual: perhaps the consequent is better explicated as the stronger ‘someone would justifiably believe a true proposition on grounds that are not sufficient for knowledge’ (cf. Sosa 2017). The third questions whether Hospital is rightly taken as the input for the Gettier-reasoning: perhaps there is a gap between it and the intended extension thereof that the philosopher successfully communicates (cf. Ichikawa and Jarvis 2013, Ch.8). Clearing this gap might seem a job for a general theory of communication.

Anyway, the account has advantages that refinements should arguably preserve. *Fit with pre-theory:* the account echoes a pre-theoretic description of participating in a Gettier thought experiment: the given text is a springboard for imagining a scenario that one judges to have certain epistemic features. *Non-exceptionalism:* understanding reality-oriented imagination as a form of simulation that bears on the epistemology of counterfactuals aligns with developments in cognitive science and psychology.^12^ Similar remarks apply to mindreading.^13^ Moreover, counterfactual, possibility and epistemic judgments are ordinary phenomena with a plausible evolutionary purpose.^14^*Possibility of success:* the argument from **W1** and **W2** to **C1** and **C2** is valid (on standard semantics). Further, general skepticism about such premises balloons into an implausible skepticism about everyday modal and counterfactual claims (cf. Williamson 2016a, b). In particular, typical Gettier cases seemingly evoke mundane possibilities and everyday epistemic notions. *Possibility of defeat (i.e. fallibilism):* Since ordinary modal, counterfactual and epistemic judgments are fallible, Gettier-reasoning is predicted to be fallible. No appeal is made to infallible ‘modal vision’, ‘rationalistic intuition’, or ‘raw conceptual competence’ (cf. Bealer 1998; BonJour 1998; Bealer 2002; Sosa 2007). This accommodates skepticism about applications of MoC where far-fetched possibilities are evoked or subjects lack requisite conceptual competence or background knowledge. (Compare Hospital to thought experiments that suspend the laws of nature or mention zombies.) Thus, *moderate modal skepticism* is accommodated, à la van Inwagen (1998). As Williamson puts it, “we are more reliable in evaluating some kinds [of counterfactuals] than others. [...] We may be correspondingly more reliable in evaluating possibility of everyday scenarios than of ‘far-out’ ones, and extra caution may be called for in the latter case” (2007, p. 164).

Further alignment with Macherian commitments is now evident. Assuming that the experimental results collected in Machery (2017) indicate that epistemic peers are genuinely disagreeing when confronted with philosophical cases, a non-exceptionalist account of MoC must apparently accommodate blameless error, i.e., fallibilism. Further, Machery endorses moderate modal skepticism. Explicitly, Machery (2017, 6.1.1) advocates skepticism towards (what he calls) *modally immodest philosophical theories*: theories committed to ambitious metaphysical necessities of peculiar philosophical interest. In support, Machery (2017, 6.2) argues that stress-testing such theories requires an ability we lack: to reliably survey unusual, atypical, and remote possibilities. Thus, his advocacy of modal modesty is grounded in a moderate modal skepticism, which he in turn grounds in MoC’s purported unreliability.

We draw two main morals. First, the basic commitments of the Williamsonian F-line and Macherian X-line are largely complementary. (sect. 3 exploits epistemic non-exceptionalism and the general reliability thesis; Sect. 4 revisits moderate modal skepticism.) Second, assuming these commitments, the demographic data reported by Machery et al. (2017, 2018a, b) and the account of MoC in Williamson (2007) render it eminently plausible that Hospital-like Gettier cases induce reliable judgment.

## Macherian pessimism

At this point, it might be puzzling how a Macherian could be pessimistic about the reliability of Gettier. Two arguments for pessimism about MoC can be extracted from Machery (2017). In this section, we explicitly apply such arguments to Gettier, and respond.

- *Worrying data.* Judgment in response to Gettier is significantly influenced by *mere* presentation: in particular, framing (Machery 2017, Ch. 2). Furthermore, particular presentations cannot be singled out as promoting accurate judgment. Thus, the Gettier judgment should be rejected as unreliable across the board.
- *Philosophy is disturbing.* Relative to traditional philosophical aims, philosophically interesting cases generally have *disturbing characteristics* that promote unreliable judgment (Machery 2017, Ch. 3). Furthermore, Gettier is no exception: Gettier cases invariably have (at least) one of these characteristics. Thus, the Gettier judgment should be rejected as unreliable across the board.

In response to the first, we *conditionally* deny the second premise: it is reasonable (given epistemic non-exceptionalism) to take *certain* Gettier cases as evincing accurate judgment, *if* there aren’t independent reasons to think Gettier cases are intrinsically disturbing. The second argument, we suggest, is thus the more basic of the two. In response to it, we again deny the second premise: Gettier cases don’t characteristically exhibit any of the disturbing characteristics identified by Machery. We elaborate below.

### Gettier and framing

Does the worrying data cast doubt on the reliability of Gettier-reasoning? To focus the discussion, we concentrate on the data issued by Study 2 of (Machery et al. 2018b).^15^ Here, 85% of respondents judge that Paul in Hospital has the impression that he knows, but doesn’t know; while only 63% of respondents judge similarly for the agent in Clock, a second Gettier case. Clock is a variant on the classic case due to Bertrand Russell. (Basically: a stopped clock happens to read 4 ’o clock on its face. At 4 ’o clock, a hapless agent observes the clock face and thereby forms a belief about the time.) What to conclude?

We doubt the right conclusion is that Gettier cases evoke significantly non-uniform or unreliable judgment, for this requires an unmotivated inductive step. The class of Gettier cases is large, varying over possible epistemic structures and narrative details. Absent an argument that our sample (Hospital and Clock) is representative, nothing rules out, for instance, that the vast majority of Gettier cases induce the singular Gettier judgment at a rate akin to Hospital, with Clock an outlier.

The conclusion is in doubt even if one grants the sample is representative, for it isn’t clear that the data exhibits a framing effect in the first place. A framing effect is exhibited by two cases when (i) there is a statistically significant difference in how subjects respond and (ii) the cases differ *only* in superficial narrative details: with respect to philosophically relevant structure, they are equivalent. Let’s grant that Hospital and Clock both deserve the title ‘Gettier case’. However, Starmans and Friedman (2012) and Turri et al. (2015) caution that Gettier cases vary significantly in underlying epistemic structure. Hospital and Clock exemplify this. In Hospital, the agent believes a proposition (‘My wife is in hospital’) on the basis of a presumed truthmaker (she was admitted to University) that differs substantially from the actual truthmaker (she was admitted to Metropolitan). Clock doesn’t share this feature. Further, the nature of the defect in the agent’s information source differs. In Hospital, the agent consults a device (a call to the hospital) that is (known to be) generally reliable with respect to the salient domain (admittance facts), but is, as a matter of (bad) luck, misleading in this one instance. In Clock, the agent consults a device (the stuck clock) that is (surprisingly) highly unreliable with respect to the salient domain (time facts), but is, as a matter of (good) luck, accurate in this one instance.

The conclusion is doubtful even if one grants the sample is representative *and* issues a framing effect. Machery (2017, p. 104) offers the following criterion for judging unreliability: “the judgments elicited by a given case are unreliable provided that they are influenced by at least a demographic variable or a presentation variable and provided that this influence is large [enough]”. Note, however, that Machery (2017, sect. 3.3.1, p. 108) doesn’t think it suffices that the influence count as ‘large’ in terms of standard benchmarks from psychology. To see why, first note with Machery (2017, p. 46) that we are concerned with cases where “the dependent variable is a percentage (e.g., the percentage of people agreeing that the character does not know the relevant proposition in the situation described by a Gettier case)”. Machery (2017, pp. 45–47) deems the independent variable’s effect size as ‘large’, relative to standard benchmarks, when the absolute difference between the percentages under two conditions exceeds 30%. Let’s say, in this case, that the variable’s influence is *significant*; assuring one that the observed effect doesn’t merely reflect noisy data. (To illustrate: for Hospital and Clock, the difference in percentage is 22%, indicating only ‘moderate’ significance.)^16^ However, ‘significance’ is then neither necessary nor sufficient for concluding that the population’s judgment is unreliable. Consider sub-populations A and B, each making up 50% of the total population. If 100% of A-respondents and 70% of B-respondents answer ‘yes’ to polar question Q, then the influence of sub-population membership is significant, but, overall, 85% of the population answer ‘yes’. If the correct answer is unknown, we can merely conclude that the population is *either* largely reliable on Q *or* largely unreliable. Further, if 52% of A-respondents and 48% of B-respondents answer ‘yes’, the difference in response is not significant, but the average response matches chance. The population is, on average, unreliable.

Thus, Machery (2017, sect. 3.3.1) proposes we attend to *average response*:^17^ a variable has a large enough effect for determining unreliability when, in the aggregate (across different values of the variable), the distribution of responses is substantially mixed, i.e., the probability of any given response is sufficiently close to chance. That is, when the influence of the variable is accounted for, disagreement is stark.

To illustrate: suppose that half the population are political conservatives and half are political liberals. Suppose that 100% of conservatives answer ‘no’ to ‘Is global warming real?’, while 100% of liberals answer ‘yes’. Thus, the distribution of ‘yes/no’ answers is 50/50. One concludes: the effect size of the (pernicious) variable of political affiliation is large enough to conclude unreliability, since it produces widespread disagreement in the aggregate. (Further, if we don’t know which of ‘yes’ or ‘no’ is right, and we cannot assume that one sub-population has special competence on the issue, then we cannot identify which sub-population has accurate judgment, so cannot ignore the overall unreliability of the population’s judgment.) Second example: suppose that 80% of conservatives answer ‘yes’ to ‘Is global warming real?’, while 100% of liberals answer ‘yes’. Then the probability that a random member of the population will answer ‘yes’ is 90%: significant agreement is exhibited. Hence, we shouldn’t take the effect size as large enough (despite a 20% difference between groups) and shouldn’t conclude that the population’s aggregate judgment is unreliable.

Now compare Hospital and Clock. Here, the aggregate probability of a certain response is presumably calculated as the probability that a random member of the population gives that answer after being assigned Hospital or Clock with a coin flip.^18^ If the experimental data is representative, the probability that ‘mere impression of knowledge’ is chosen over ‘knowledge’ is thus 74%. This represents notable agreement. (Machery presumably agrees: compare the ‘room color’ example discussed by (Machery 2017, p. 104).) So why conclude significant unreliability, rather than lightly tempering one’s credence that ‘mere impression of knowledge’ is the right answer?

Turn to our main argument, which is maximally concessive to Machery. Let’s grant that the data indicates that Gettier-reasoning is significantly unreliable in the aggregate. Nevertheless, a question remains as to the exact conclusion this warrants.

- *Option 1.* Judgment in response to Gettier cases is not terribly reliable in the aggregate.
- *Option 2.* While judgment in response to Gettier cases is not terribly reliable in the aggregate, judgment relative to certain Gettier cases (or presentations thereof) is reliable.

Option 2 is a stronger hypothesis, and better explains the overall data. As noted previously, there is independent evidence that judgment induced by certain (presentations of) Gettier cases yields significant agreement across diverse demographics (Machery et al. 2018a, b). This uniformity is explained by Option 2 and left mysterious by Option 1. Certainly, if Gettier-reasoning were invariably unsystematic, then robust agreement on any particular Gettier case would be extremely surprising. So Option 2 should be accepted over Option 1, on abductive grounds.^19^

*A fortiori*, one shouldn’t *suspend* judgment on the question of reliability (as a moderate restrictionist might advocate).^20^ There is a good reason to take judgment induced by certain cases as reliable: this best explains a striking regularity.

But what of the possibility that significant agreement on a particular Gettier case indicates that our judgment is *systematically inaccurate* on that case? If this were a serious possibility, then Option 3 could be deployed to explain the data, on a par with Option 2.

- *Option 3.* Judgment in response to Gettier cases is not terribly reliable in the aggregate, and judgment relative to certain Gettier cases (or presentations thereof) is systematically inaccurate, generating an *epistemic illusion*.

However, an epistemic non-exceptionalist should *not* take Option 3 seriously without specific support for it over Option 2. Absent specific evidence that a certain (presentation of a) Gettier case corrupts judgment, she observes a basic confidence in ordinary judgment. If the case generates widespread agreement (relative to a large and diverse population of individuals), the presumption should be that ordinary judgment has here largely yielded accurate (‘expert’) judgment, as is typical for ordinary cases. Compare a toy example: suppose that half of the population of *climate scientists* are liberals, half are conservatives. It turns out that 98% of the former answer ‘yes’ to ‘Is climate change real?’, compared to only 60% of the latter. The uniformity among liberals is striking. Should we posit that their judgment is systematically inaccurate (wholly corrupted by political brainwashing)? This is excessively skeptical, in the absence of specific evidence. The normal presumptions stand until defeated: a scientist’s judgment is normally expert, and expert judgment generally converges. Thus, the best explanation for the uniform liberal judgment is that it is accurate: the liberal experts judge exactly as we would expect experts to judge (striking consensus); while the conservative experts judge as we would expect experts to judge under the influence of disturbing factors (a mixed response).^21^

*Is* there independent reason to think that Gettier-reasoning typically exhibits peculiarities that jeopardize ordinary judgment? Were the answer ‘yes’, Option 3 would be live. We’ll argue ‘no’ with respect to the ‘disturbing characteristics’ proposed by Machery (2017).

### Is Gettier disturbing?

Machery (2017, Ch. 3.5) argues that philosophically interesting cases typically have one of three *disturbing characteristics* that promote unreliable judgment:

- *Entanglement.* Judgment of the case is influenced by its superficial content. That is, arbitrary narrative details (that merely render the case concrete and vivid) influence our judgment, though they have no real bearing on the issue the case is intended to investigate.
- *Unusualness.* The case describes an unusual situation, relative to the demands of ordinary life. Ordinary life doesn’t offer opportunities to exercise judgment in such situations (not even unrealized opportunities), so we cannot assume ordinary judgment is primed for them.^22^
- *Atypicality.* The case pulls apart properties that generally co-occur in ordinary life, sabotaging the heuristics of ordinary judgment and encouraging *ad hoc* responses.

It is explicable that philosophically interesting cases tend to have these features. Philosophy investigates phenomena that, while familiar and fundamental, puzzle us on close inspection. We engage in philosophical reflection precisely because we struggle to delineate core features. It is therefore difficult to guard against (or correct) entanglement. Further, philosophical theories often target necessary truths, with rival theories often agreeing on everyday cases. Such theories can only be stress-tested with unusual or atypical cases.

We discuss each disturbing characteristic in relation to Gettier, in turn.

### Entanglement

We grant that philosophical cases face a *threat* of **Entanglement**: it is hard to rule out that any particular judgment is subject to entanglement. Further, we tentatively grant that there is specific evidence of entanglement in the case of Gettier: as noted, Machery et al. (2018b) report that responses to certain Gettier cases are influenced by merely presentational factors.^23^

Given the general reliability thesis, one must deny that the mere threat of entanglement casts doubt on the reliability of Gettier-reasoning. If it did, there would be similar grounds for doubting the reliability of countless ordinary epistemic judgments: the latter seem no less susceptible to entanglement. You see Sam reading the headline of today’s New York Times. The headline states that Clinton lost the election. Sam is, in your experience, an affable and reasonable person. Further, you are aware of the Times’ reputation for journalistic excellence and find it an enjoyable read. You judge (rightly) that Sam thereby knows that Clinton lost the election. But the threat of entanglement is present. Absent general confidence in ordinary epistemic judgment, nothing rules out the possibility that one’s judgment has here been influenced by epistemically irrelevant features of the situation (say, one’s warm feelings for Sam or the New York Times). As usual, it is difficult to *exactly* delineate the features of the situation that make the knowledge ascription reasonable, so a more cautious assessment of Sam’s epistemic state is elusive.

What of the specific evidence that presentation influences Gettier-reasoning? We reiterate our conclusion from Sect. 3.1: given epistemic non-exceptionalism, the best explanation of the *overall* data is that only *certain* Gettier cases (or presentations thereof) are likely entangled. This suggests that adverse presentation effects can be ameliorated by a judicious selection of presentational features (and that experimental philosophy provides useful tools for identifying them). Call those Gettier cases that elicit markedly stable judgment *sober*. Going forward, we focus on such and assume Hospital is among them.

### Unusualness

That Gettier cases are unusual has initial support, as Weinberg (2017, sect. 3) notes. Anecdotally, philosophy students find them surprising on first encounter. Some need help to grasp their structure: rushing their introduction seems a pedagogical error. Experimentally, Turri (2013) reports that judgments about Gettier cases converge much more readily if their structure is presented with extra perspicuity. There is evidence, then, that Gettier cases don’t regularly emerge for evaluation in ordinary life, and ordinary faculties aren’t always primed to notice and properly assess them.

It doesn’t follow that (Hospital-like) Gettier cases are intrinsically disturbing. To show this, we decompose Hospital.

- *Component 1:* *Justified belief without ‘sensitivity’*.^24^
  Starting to worry that something might have happened to his wife, Paul Jones decided to call some local hospitals to ask whether any patient by the name of “Mary Jones” had been admitted that evening. At the University Hospital, the person who answered his call confirmed that someone by that name had been admitted with major but not life-threatening injuries following a car crash. Paul grabbed his coat and rushed out to drive to University Hospital. As it turned out, the patient at University Hospital was not Paul’s wife, but another woman with the same name.
- *Judgment.* Paul didn’t come to know anything about his wife via the call, but it led him to justifiably/reasonably/blamelessly believe she was hospitalized.
- *Component 2:* *True belief.*
  Paul’s wife had a heart attack as she was leaving work, and was actually receiving treatment in Metropolitan Hospital, a few miles away.
- *Judgment.* Paul had a true belief if he believed his wife was hospitalized.

Component 1 yields, by itself, a key judgment: Paul’s ignorance. Strikingly, the truth value of ‘Mary Jones is in hospital’ needn’t be specified for this judgment to be apt. A tempting conclusion: the truth value is *irrelevant*. A ready explanation: misleading appearances aside, University’s admission roster holds no information about Paul’s wife, and sources that are uninformative about X don’t induce knowledge about X.

The general phenomenon is familiar and mundane. Suppose Ann asks Bob, a trustworthy person: “Does Carol eat meat?” Bob sincerely replies: “No, Carol is vegetarian. She told me so”. However, Ann and Bob are speaking at cross purposes: Ann is talking about *Carol Jones*; Bob about *Carol Smith*. Indeed, he doesn’t know anything about (doesn’t hold information concerning) the dietary preferences of Carol Jones. Ann might thereby reasonably believe Carol Jones is vegetarian, but this isn’t knowledge; Bob didn’t communicate any knowledge *about Carol Jones*. Whether or not Carol Jones is in fact vegetarian seems irrelevant to this mundane assessment. Another instance: Ann asks Bob: “Do all the conference speakers eat meat?” Bob sincerely replies: “No, one of them told me she is vegetarian”. However, Bob is talking about Carol Smith: he mistakenly believes she is a conference speaker. Indeed, he doesn’t know the dietary preferences of any conference speaker. Ann forms a reasonable belief that not every conference speaker eats meat. This isn’t knowledge; Bob didn’t have any to communicate. Whether any speaker is in fact vegetarian is irrelevant.

Further, Components 1 and 2 are, on their face, simple and mundane. Assuming the general reliability thesis, ordinary judgment is primed for such circumstances: absent defeating considerations, our assessment is trustworthy.

Of course, situations akin to Component 1 and 2 might occur infrequently. If so, they are unusual, in a straightforward sense. Does *this* defeat default confidence in our immediate judgments? No—it rather illustrates that low probability events can be mundane and, therefore, apt for reliable judgment. As Williamson (2016b, sect. 2.3) observes, to assume that low probability events invariably disturb ordinary judgment is markedly skeptical: just about any situation is of low probability under the right description. Indeed, it is evident that ordinary judgment *doesn’t* collapse in the face of rare/unexpected events: if it did, we would be severely impeded in ordinary life.

If there is anything *notably* intriguing and unusual, it is the *combination* of Component 1 and 2. Mere combination can introduce two complications: lowered probability and heightened complexity. But, again, ordinary judgment isn’t so brittle as to collapse in the face of lightly improbable combinations of ordinary situations. Sam reads in the New York Times that Clinton lost the election. Conclusion: she knows Clinton lost. Blake reads in the New York Times that Clinton lost the election. Conclusion: she knows Clinton lost. Coincidentally, they read exactly the same copy of the NYT (at a certain doctor’s waiting room; they both fell sick that day). We wouldn’t and shouldn’t retract our initial judgments of knowledge simply because of this coincidence. Rare combinations of mundane elements are sometimes mundane. Similar remarks apply to complexity introduction. We face complex situations in ordinary life (e.g. a busy city street). Navigating them requires skills in complexity management: selective attention and careful bookkeeping. An agent that lacks these is again severely impeded, certainly in high stakes situations. So, if complexity *invariably* disturbed ordinary judgment, the general reliability thesis would be undermined. Of course, Turri (2013) provides *prima facie* evidence that the complexity of some Gettier cases disturbs ordinary judgment. Unsurprisingly, this is ameliorated with a careful presentation (explaining why introducing Gettier cases to students requires care). Anyway, the experimental results indicate Hospital doesn’t fall prey to such disturbance.

At any rate, even if the combination of Component 1 and 2 *could* lead to confusion, a simple strategy safeguards accuracy: be careful to judge the components individually and then conjoin the judgments. Could the combination of Component 1 and 2 somehow defeat the considerations that render the corresponding judgments individually apt? This strains credulity: the respective considerations seem decisive. Again, consider Component 1: it seems obvious that knowledge about X cannot accrue from a source that carries no information about X—no matter the circumstances of X.

So, is Hospital *disturbingly* unusual? This conclusion isn’t licensed simply because it involves rare events or relative complexity. It seems a harmless combination of simple mundane elements: ordinary judgment is presumably here expert, a matter of merging individual judgments about Component 1 and 2. No experimental result defeats this presumption.

Weinberg (2017, sect. 3) proposes a more subtle reason to take (the simple elements of) Gettier cases as disturbingly unusual: they hinge on information about the ‘specific inferential pathways’ taken by the Gettierized agent. (He continues: “And it seems to me we only in the rarest of circumstances are in a situation to [furthermore] know that [the agent’s] belief might be true, while also being aware of a range of possible truthmakers for that belief” (idem., p. 265).) Weinberg suggests there is a profound lack of such information in ordinary life. In Gettier (1963), an agent uses disjunction-introduction to infer ‘Jones owns a Ford or Brown is in Barcelona’ from ‘Jones owns a Ford’, where ‘Brown is in Barcelona’ was randomly selected. It is hard to think of mundane situations where someone transparently reasons like this.

However, to claim mundane situations never yield information about ‘specific inferential pathways’, broadly understood, is to exaggerate. Ordinary speakers often report their reasoning for evaluation. Ann: “Someone in the office is vegetarian”. Dave: “How do you know?” Ann: “Bob is Carol’s good friend and told me she is vegetarian”. One judges: Ann believes that someone in the office is a vegetarian, on the basis (of her belief) that Carol is. One judges: she knows the former if she knows the latter, which hinges on whether Bob knew it. This is exceedingly mundane. (As is observing that Ann’s belief that someone is vegetarian might be true, and could be made true by multiple possible situations.)

Grant that disjunction introduction yields strange reasoning. Not all Gettier cases involve such strangeness. Another classic case from Gettier (1963) hinges, less artificially, on existential-introduction. Hospital induces a perfectly ordinary judgment about an agent’s reasoning: Paul believes his wife was admitted to University, on the basis that her name is on the admission roster.

In short, Hospital might be unusual, but, assuming general reliability, we shouldn’t take it as *disturbingly* unusual: unusual in any sense that undermines ordinary judgment. To generalize: absent specific defeat, cases in this structural family shouldn’t be counted by an epistemic non-exceptionalist as disturbingly unusual if constructed from simple mundane elements, presented with perspicuity, and assessed with care.

### Atypicality

Turn to **Atypicality**. Machery worries about situations where there is a package of features, e.g., *a*, *b*, *c*, that typically indicates $\Phi$, and ordinary judgment exploits this as a mere *heuristic*. Thus one shouldn’t conclude from our ordinary practice that any of *a*, *b* or *c* is *necessary* for the truth of $\Phi$, nor that ordinary judgment is reliable when the package is pulled apart. Hence, philosophically interesting cases that fracture the package yield dubious judgments. In the case of Gettier, the typical package ‘truth + justification + sensitive belief’ indicates knowledge, and serves as a heuristic for ordinary judgment. (We grant these claims.) But Gettier pulls sensitivity (whatever it is) apart from truth and justification. Hence, the worry goes, Gettier induces unreliable judgment.

In response, two points: (i) splitting a typical package doesn’t *necessarily* lead to unreliable judgment; (ii) Gettier plausibly investigates exactly this sort of split (i.e., where reliability is not undermined).^25^ To see (i), consider: it is easy to think of ordinary situations where justification is present without truth. Here, a typical package is pulled apart. But we shouldn’t conclude that judgment in these situations is unreliable, since lack of truth is an ordinary, decisive marker of ignorance (as Machery 2017, sect. 3.6.3 notes). In support of (ii), we suggest that lack of sensitivity (whatever exactly it is) is analogous to lack of truth: an ordinary, decisive marker of ignorance. Again consider Component 1: a mundane situation where we judge an agent as ignorant, given a lack of sensitive belief. Despite being hard to make precise, the rationale for this judgment is again easily gestured at. Though Paul is unaware of it, the admission roster issues misleading evidence concerning his wife. Indeed, misleading appearances aside, it clearly carries *no* information about his wife. Agents that form beliefs on the basis of a (relevantly) bereft information source don’t thereby acquire knowledge. Compare: an agent that forms beliefs about a celebrity’s lifestyle on the basis of The National Enquirer doesn’t thereby accrue knowledge. The badness of the source is decisive: it doesn’t (seem to) matter if the belief happens to be true or if the agent has somehow been convinced to consider the National Enquirer trustworthy.^26^

In short, Gettier cases like Hospital might be atypical, but, assuming general reliability, one shouldn’t conclude a *disturbing* atypicality.

In sum: we see no compelling reason for an epistemic non-exceptionalist to take disturbing characteristics as intrinsic to (or typical of) Gettier cases in Hospital’s structural family.

## Upshot for Machery’s master arguments

### Radical restrictionism

*PwPB* defends a severe conclusion: philosophers should abandon the traditional method of cases. Machery reasons inductively, using an inductive step:

If the judgments elicited by most of the philosophical cases that have been examined by experimental philosophers are unreliable, then the judgments elicited by most philosophical cases are plausibly unreliable. (2017, p. 102)

He offers three lines of support for this claim:

1. The tested cases are typical examples of philosophical cases: they “possess many of the properties many philosophical cases possess” (2017, p. 109).
2. “[The tested cases] are canonical. They are famous, and, consciously or unconsciously, they function as templates or paradigms when philosophers write novel cases” (2017, pp. 109-110).
3. Philosophically interesting cases typically posses the *disturbing characteristics* discussed above, so its members are generally relevantly similar to the cases that have been tested (2017, sect. 3.5).

On this basis, the tested cases are claimed to be *representative* of the class of philosophically interesting cases.^27^

Should we accept the inductive step? We proceed on the assumption that our previous arguments have been successful: Gettier cases needn’t be taken to generally exhibit disturbing characteristics; Gettier-reasoning (applied to sober cases) induces reliable judgment; and naturalists needn’t find this mysterious, as a Williamsonian analysis illustrates. In particular, we assume this for Gettier cases with the underlying epistemic structure of Hospital, including those of Gettier (1963). This puts pressure on the inductive step.^28^ Gettier cases are clearly philosophically interesting. They aren’t intrinsically disturbing. They are typical. They are (especially) canonical: few thought experiments (even limiting ourselves to Hospital’s class) have been as influential or elicited as much consensus among philosophers. Certainly, it is rash to assume that cases that are controversial among philosophers (precisely because they plausibly disturb ordinary judgment) better represent the broad class of philosophically interesting cases.^29^ In short, even if 1 and 2 are true, 3 and the inductive step shouldn’t be casually accepted: what rules out that philosophically interesting cases are frequently akin to sober Gettier cases like Hospital?

Even if one grants Machery’s inductive step (and that most tested cases induce unreliable judgments), one can resist his severe conclusion. For he requires another conditional: if most philosophically interesting cases induce unreliable judgment, then MoC should be abandoned. But Gettier, it seems, showcases a class of cases for which MoC proves effective, with significant philosophical benefits in tow (as its influence attests). This success should be preserved and emulated. The experimental results are a signal for caution and reform. MoC shouldn’t be abandoned, but recognized as fallible and utilized with discipline (and experimental checks). Gettier (Hospital-like cases in particular) represents a paradigm towards which MoC can and should aspire.

Machery (2017, sect. 5.6) is skeptical about the prospects for reforming MoC. Further, he anticipates objections to his inductive argument. He writes:

Nor is it an objection that some philosophical cases may not possess any disturbing property. The claim is not that every philosophical case elicits a cognitive artifact or diverse responses, but that the kind of case philosophers use for dialectical purpose tends, non-accidentally, to elicit cognitive artifacts or a diversity of responses. (2017, p. 183)

Our own objections don’t rest merely on the possible existence of philosophical cases that aren’t disturbing: we are *not* fallaciously proposing that a single counter-example undermines a statistical or generic claim. Our key claim is that certain *typical* and *canonical* philosophical cases don’t possess disturbing properties. In this connection, we emphasize that Machery doesn’t deploy vanilla statistical-inductive reasoning: he doesn’t base his conclusion that most philosophical cases elicit unreliable judgments on a (demonstrably) *random* and *suitably large* sample of tested philosophical cases (or, indeed, of tested typical and canonical cases). Nor does he establish the *relative degree* of typicality or canonicity for various philosophical cases, as would be essential for evaluating the plausible hypothesis that Hospital-like Gettier cases typify an especially large bulk of philosophical cases. Thus, he hasn’t established that his sample of tested cases warrants generalization to most or all philosophical cases; nor that Gettier isn’t by itself a significant success story for MoC.

Machery continues:

Nor is it compelling to respond that the advice to suspend judgment remains inapplicable until there is clear-cut evidence about what cases exactly are impugned by experimental-philosophy studies. First, we have provided reasons to believe that disturbing cases prime unreliability and disagreement. Second, even if we were unsure about how broadly to suspend judgment, we should still suspend judgment in response to all the cases in contemporary philosophy (except those known to be immune to demographic and presentation effects) because the cases examined by philosophers are typical and canonical. Similarly, if we find that some eggs are contaminated with Salmonella, we would stop eating eggs sold by the brand selling them, even if it is unclear whether all eggs are contaminated. (2017, p. 183)

In issuing a blanket ban on new applications of MoC (though he grants the possibility of cases that are immune to serious demographic and presentation effects), Machery underestimates our ability to (reasonably, defeasibly) discriminate between philosophical cases that are likely or unlikely to induce reliable judgment. Compare the debate induced by the proposal in Weinberg (2007) that epistemic judgment about philosophical cases isn’t sufficiently *hopeful*: we lack robust error-detection mechanisms for regulating it. Ironically, Machery (2017, Ch. 3) convincingly defuses generic worries about hopefulness. Further, studies reported by Wright (2010, 2013) suggest that ordinary respondents reliably register the presence of instability/unreliability in their epistemic judgments.^30^ Machery has himself identified a rough but promising list of features that problematic cases typically exhibit: namely, the disturbing characteristics (entanglement, unusualness, atypicality). If such characteristics are lacking (as far as one can tell), an epistemic non-exceptionalist assumes that ordinary judgment is primed to rule accurately on what appears to be an ordinary case. This assumption can, of course, be defeated by experimental investigation. Granted, some disturbing characteristics may be hard to discern: entanglement, for instance. However, our reservation in taking the mere threat of entanglement too seriously (Sect. 3.3) is again pertinent. Other characteristics seem easier to spot: modally exotic cases involving philosophical zombies or evil demons seem easily distinguished from relatively mundane cases like Hospital.

So the Salmonella analogy is inapt. Contrast a second case of egg contamination. In the summer of 2017, The Netherlands experienced a large scale contamination of eggs with *fipronil*, a poisonous insecticide (NOS 2017a, b). The level of fipronil was so high in certain clusters of eggs that those eggs were inedible. But the National Health Organization merely advised people to ‘proceed with caution’ when consuming eggs, rather than halt consumption altogether. This was sensible: it was reasonably clear which eggs were contaminated. Indeed, a serial number is printed on every egg, and the Dutch National Health Organization was able to release a list of numbers for eggs that were reasonably suspected to be infected.

The same advice applies to MoC: a naturalist should proceed with caution, but to discontinue MoC entirely is an overreaction to the data.

### Modal modesty

Macherian pessimissm about Gettier should be unconvincing to both Williamsonians and Macherians, in virtue of common ground: epistemic non-exceptionalism. The Williamsonian F-line gets a better handle on Gettier: suitably mundane cases like Hospital deploy ordinary possibility, counterfactual and epistemic judgment in the production of substantive philosophical knowledge. So much for the claim that (traditional) MoC has not or cannot yield substantive philosophical conclusions, and should be shelved.

However, Machery (2017, Introduction) describes his critique of MoC as a detour on the way to his *main* conclusion that “resolving many traditional and contemporary philosophical issues is beyond our epistemic reach” (p.1); in particular, “modally immodest issues cannot be resolved, and modally immodest philosophical views [cannot be] supported” (p. 3). Philosophers, he worries, often pursue theories of knowledge, mind, personal identity, right action and free will that target ostentatious claims of metaphysical necessity. Machery (2017, sect. 6.1.1) offers this argument:

**M1.**: Many central philosophical issues are about metaphysical necessities, and resolving these issues requires establishing these necessities.
**M2.**: Philosophers must appeal to unusual and atypical philosophical cases to establish these metaphysical necessities.
**M3.**: We should suspend judgment about the situations described by current philosophical cases and, more generally, by unusual and atypical philosophical cases.
**M4.**: There is no other way of learning about the pertinent metaphysical necessities and possibilities.
**MC.**: Hence, there are many philosophical issues that we cannot resolve.

We reject **M3**: Hospital counts as a ‘current philosophical case’ that is, broadly speaking, unusual (Sect. 3.4) and atypical (Sect. 3.5), yet apt for judgment. Since Hospital represents a canonical and typical class of cases, **M3** shouldn’t even be accepted *generically*.

However, a nearby argument is harder to dismiss. Gettier-reasoning is typically mundane and well-supported by empirical studies. This cannot be said for a large swathe of tested philosophical cases: *Truetemp, Switch, Transplant, Society of music lovers*, etc. Unlike Hospital, these don’t strike us pre-theoretically as (unlucky but) mundane: they are unusual or atypical in a plausibly disturbing sense. Indeed, empirical investigation reveals serious demographic and presentation effects (Machery 2017, Ch. 2). Suppose these cases are indeed canonical and typical examples of a larger class of *exotic* philosophical cases (in contrast to *mundane* philosophical cases). Indeed, they largely belong to a salient sub-class: modally *remote* cases, instantiated only in suitably ‘distant’ possible worlds. One may then deploy an inductive argument (analogous to but more modest than that in Sect. 4.1): MoC applied to exotic philosophical cases is unreliable. This supports:

M3*.: We should suspend judgment about the situations described by exotic (e.g. remote) philosophical cases.

Here is a variant of **M2**:

M2*.: Philosophers must appeal to exotic (e.g. remote) philosophical cases to establish these metaphysical necessities.

Replacing **M2** with M2* and **M3** with M3* yields a Macherian argument for **MC** that is untouched by our foregoing critique. The tentative neo-Macherian moral: philosophers ought not abandon (substantive uses of) MoC, but limit it to (putatively) mundane cases that cannot support especially ambitious, modally immodest metaphysical theses.

This argument deserves careful scrutiny. It aims to support a moderate modal skepticism that is subtly different from a more familiar form (cf. van Inwagen 1998).^31^ The Williamsonian account of MoC (Sect. 2) helps to draw the distinction. The familiar form worries about the gap between merely considering/imagining a concrete remote case and having established its possibility: premise **W1** in the reconstructed MoC reasoning is questioned. The Macherian form worries about our accuracy when applying ordinary judgment to remote cases: **W2** is questioned. Thus, suspicion is raised about judgments about knowledge, right action or free will in response to *clearly possible but remote* cases. Counterpossible conditionals (i.e., counterfactual conditionals with impossible antecedents^32^) also highlight the contrast, if impossible antecedents are both remote and support only some consequents. Van Inwagen-style modesty worries about whether and when we can rightly *identify* a counterpossible conditional. Macherian modesty worries about whether and when we can rightly assess its *truth*.

So, a version of Machery’s core argument for rejecting modal immodesty survives our critique. Detailed evaluation is for future work. Our tentative conclusion: a promising and prominent naturalistic programme spanning the F-line and X-line is plausibly committed to *both* the reliability of typical Gettier-reasoning *and* modally modest philosophy.

## Footnotes

1 Our characterization doesn’t entail severe naturalism: for instance, that philosophers should limit themselves to straightforwardly empirical methods.

2 Cf. Sosa (2007).

3 Malmgren (2011) and Ichikawa and Jarvis (2009, 2012, 2013) are F-liners that accommodate rationalism.

4 Williamson (2014) explicitly rejects the ‘naturalism’ label. Naturalists, he complains, tend to equivocate between an unattractively severe position and a harmless but vacuous one. However, Williamson (2007) and Machery (2017) occupy an attractive middle ground. At any rate, we’re ultimately more interested in their shared commitments than the choice of label.

5 Machery (2017, Ch. 4) develops a second argument for abandoning MoC, as follows. Experimental investigation reveals that philosophical thought experiments yield inconsistent judgments among epistemic peers. If the disagreement is real, philosophers ought to suspend belief on the deliverances of MoC, lest they be dogmatic. But perhaps the disagreement is *merely* apparent: philosophers and non-philosophers differ in their interpretation of the cases. In this case, philosophers ought to focus on which interpretation reflects the most significant issues, to avoid over-emphasizing merely parochial concerns. Concerning Gettier, one can respond as follows: as we shall discuss, experimental results do *not* indicate robust disagreement among epistemic peers on the status of (certain) Gettier cases (Machery 2017, sec. 4.1.4 explicitly concedes this). Machery’s dilemma plausibly doesn’t get off the ground.

6 Vetter (2017) helpfully explicates the Williamsonian ideology.

7 The studies test both respondents’ inclination to choose between a knowledge attribution and a straightforward ignorance attribution, and their inclination to choose between a knowledge attribution and describing the agent as merely having the impression that they know. The latter seems to us more revealing.

8 Israeli Bedouins were an outlier; Machery et al. (2017) advise caution in light of a small sample size.

9 These are ‘apparent evidence’ cases, in the terminology of Starmans and Friedman (2012).

10 Turri et al. (2015) observe a subtlety: the singular Gettier judgment seems notably suppressed if the actual (‘replacement’) truthmaker is suitably similar to the presumed truthmaker. The divergence dissipates for presentations that help respondents track underlying epistemic structure (Turri 2013).

11 Williamson (2016a) argues that skepticism about ‘philosophical intuition’ is implausible if understood to encompass large swathes of mundane judgment. As for Machery: “[Radical restrictionism] is not a skepticism about judgment in general or, more narrowly, about the judgments concerning the topics of philosophical interest—e.g. knowledge, causation, permissibility, or personal identity” (Machery 2017, p. 7).

12 Support comes from a variety of fields. Philosophy of mind/imagination: Currie and Ravenscroft (2002), Nichols and Stich (2003), Byrne (2005, 2016), Langland-Hassan (2016). Cognitive Psychology: Goldman (2006), Nichols (2006). Cognitive Neurosciences: Van Hoeck et al. (2015). Developmental Psychology: Rafetseder et al. (2010), Walker and Gopnik (2013), Lane et al. (2016).

13 Gallese and Goldman (1998), Currie and Ravenscroft (2002), Nichols and Stich (2003), Goldman (2006), Gallese (2007). Also see Nagel (2012) and the extensive references therein.

14 See Hesslow (2002), Byrne (2005), Nichols (2006), Williamson (2007), Epstude and Roese (2008), Pezzulo (2011), Byrne (2016); and Williamson (2016a).

15 Other studies would serve equally well, though specific points might require tailoring. Machery et al. (2018b) offer evidence that Gettier is subject to order effects. Machery (2017, sect. 2.6.2) rightly points out that the effect is small. Machery et al. (2018a) observe that a certain Gettier case (the ‘Trip case’) induces a singular Gettier judgment at a markedly lower rate than Hospital, despite having a similar underlying epistemic structure. Again, the divergence is less marked than between Hospital and Clock. Turri et al. (2015) and Turri (2019) report studies indicating that Gettier cases with differing epistemic structure yield a singular Gettier judgment at different rates. Beebe and Shea (2013) report a study indicating that the moral valence of the agent’s actions affects how subjects respond to a Gettier case.

16 Additionally, when one looks at standard normal curve test from statistical power analysis to measure the effect size of “the difference between two independent proportions” (Cohen 1992, p. 157), which is gives the largest effect size of the discussed these methods, the effect size is still only medium (given $h_{h} = .85$ and $h_{c} = .63$, the arcsine transformation $\phi = .51$) (cf. Cohen 1988, Ch. 6 and Cohen 1992, Table 1). Thanks to Rob Schoonen for a helpful discussion.

17 Relatedly, Machery responds to criticism from Demaree-Cotton (2016)—who argues that he concludes unreliability too quickly - that she “does not address the issue [of effect size] from the right angle” (2017, p. 108).

18 Let $\mathtt {Pr}(K | H)$ be the probability that a random respondent selects ‘knowledge’ on the condition they were assigned Hospital; let $\mathtt {Pr}(K | C)$ be the probability that they select ‘knowledge’ on the condition they were assigned Clock. Then the aggregate probability of the ‘knowledge’ response is $0.5 \times \mathtt {Pr}(K | H) + 0.5 \times \mathtt {Pr}(K | C)$, i.e. $0.5 \times 0.15 + 0.5 \times 0.37$, i.e. 0.26.

19 Machery (2017, p. 106) claims that “it is hard to see which of the frames or which of the orders of presentation would make it more likely that people get it right about the situations described by philosophical cases”. This is exactly what we deny.

20 Notable moderate restrictionists happily concede that Gettier-reasoning can be reliable: see Alexander and Weinberg (2014) and Weinberg (2017).

21 Should we suspend judgment simply because there is significant disagreement between epistemic peers; indeed, presumed experts? (Cf. Machery 2017, ch. 4.) We say ‘no’. *Mere* disagreement between peers needn’t prompt suspension of judgment: if 98% of experts agree on a question, one should accept the consensus without hesitation, despite some dissent. Generally, it is plausible that one should calibrate one’s credence in line with the strength of consensus among experts. What’s more, there are plausibly cases where one should give more weight to certain large sub-classes of expert, e.g. as in our toy example, when the sub-class of liberal experts judge as we expect experts should judge (i.e. with broad consensus), as opposed to the sub-class of conservative experts, who judge as we expect experts to judge in the presence of disturbing factors.

22 Cf. Weinberg (2017, p. 265): the relevant sense of ‘unusualness’ needn’t “concern the frequency of the occurrence of Gettier-type situations, but the frequency of epistemic evaluations of Gettier-type situations, in which the relevant aspects of the situation are recognized and even capable of being brought into the evaluation.”

23 Though recall Sect. 3.1’s reservations in concluding too hastily that two Gettier cases are equivalent with respect to core philosophical features.

24 That is, with merely ‘apparent evidence’, in the terminology of Starmans and Friedman (2012), or ‘without detection’ in the terminology of Turri et al. (2015), Turri (2019).

25 See Williamson (2016b, sect. 2.3).

26 In the terminology of Machery (2017, sect. 3.6.3), our claim is that sensitivity is a ‘central component’ of the concept of knowledge, just as falsehood is a central component of our concept of ignorance.

27 Machery (2017, pp. 128–129) deploys a similar inductive step with similar justification in support of another argument for abandoning MoC: an argument from peer disagreement. Thus our critical remarks transfer to this second argument.

28 Levin (2019) also suggests that Machery’s inductive argument might be too quick.

29 For instance, the zebra case of Dretske (1970); the zombie case of Chalmers (1996); the ‘fake barn’ case of Goldman (1976). For clearly articulated suspicions about the force of some philosophers’ knee-jerk judgments about these cases, see van Inwagen (1998), Hawthorne (2004), Gendler and Hawthorne (2005).

30 Machery (2017, p. 122) observes that the findings in Wright (2013) show that low confidence predicts unreliable/unstable judgment, but don’t establish that high confidence predicts reliable/stable judgment. Thus, low confidence plausibly defeats the presumption that a judgment is stable/reliable. Our default trust in our (confident) judgment, meanwhile, can rest, for a Macherian, on the general reliability thesis.

31 A potential objection: **M2*** claims that philosophical cases are used to *establish* metaphysical necessities. The exact methodology isn’t obvious, however: typical philosophical cases primarily establish metaphysical *possibilities* that most obviously serve to *refute* necessity claims (by counterexample). Debates concerning necessity- or possibility-first epistemologies of modality are relevant here (cf. Hale 2003; Fischer 2016). Also, **M4** is a strong claim: a variety of methods in the epistemology of (metaphysical) modality have been proffered (cf. Vaidya 2016).

32 See Nolan (1997).

## References

[CR1] Alexander, J., & Weinberg, J. M. (2014). The “Unreliability” of Epistemic Intuitions. In E. Machery, & E. O’Neill (Eds.) *Current controversies in experimental philosophy*, chap. 8, (pp. 128–145). Routledge.

[CR2] Bealer, G. (1998). Intuition and the autonomy of philosophy. In M. R. DePaul, & W. Ramsey (Eds.) *Rethinking intuition: The psychology of intuition and its role in philosophical inquiry*, (pp. 201–40). Rowman & Littlefield.

[CR3] Bealer G, Szabó Gendler T, Hawthorne J (2002). Modal epistemology and the rational renaissance. Conceivability and possibility.

[CR4] Beebe JR, Shea J (2013). Gettierized Knobe effects. Episteme.

[CR5] Boghossian PA (1996). Analyticity reconsidered. Noûs.

[CR6] BonJour L (1998). In defense of pure reason: A rationalist account of a priori justification.

[CR7] Byrne RMJ (2005). The rational imagination.

[CR8] Byrne RMJ (2016). Counterfactual thought. Annual Review of Psychology.

[CR9] Chalmers DJ (1996). The conscious mind.

[CR10] Cohen J (1988). Statistical power analysis for the behavioral sciences.

[CR11] Cohen J (1992). A power primer. Psychological Bulletin.

[CR12] Currie G, Ravenscroft I (2002). Recreative minds.

[CR13] Demaree-Cotton J (2016). Do framing effects make moral intuitions unreliable?. Philosophical Psychology.

[CR14] Dretske F (1970). Epistemic operators. The Journal of Philosophy.

[CR15] Epstude K, Roese NJ (2008). The functional theory of counterfactual thinking. Personality and Social Psychology Review.

[CR16] Fischer B (2016). Hale on the architecture of modal knowledge. Analytic Philosophy.

[CR17] Gallese V (2007). Before and below ‘theory of mind’: Embodied simulation and the neural correlates of social cognition. Philosophical Transactions of the Royal Society B: Biological Sciences.

[CR18] Gallese V, Goldman AI (1998). Mirror neurons and the simulation theory of mind-reading. Trends in cognitive sciences.

[CR19] Geddes A (2017). Judgements about thought experiments. Mind.

[CR20] Gendler TS, Hawthorne J (2005). The real guide to fake barns: A catalogue of gifts for your epistemic enemies. Philosophical Studies.

[CR21] Gettier E (1963). Is justified true belief knowledge?. Analysis.

[CR22] Goldman AI (1976). Discrimination and perceptual knowledge. The Journal of Philosophy.

[CR23] Goldman AI (2006). Simulating minds: The philosophy, psychology, and neuroscience of mindreading.

[CR24] Hale B (2003). The presidential address: Knowledge of possibility and of necessity. Proceedings of the Aristotelian Society.

[CR25] Hawke P (2011). Van Inwagen’s modal skepticism. Philosophical Studies.

[CR26] Hawke P, Fischer B, Leon F (2017). Can modal skepticism defeat humean skepticism?. Modal epistemology after rationalism.

[CR27] Hawthorne J (2004). Knowledge and lotteries.

[CR28] Hesslow G (2002). Conscious thought as simulation of behaviour and perception. Trends in Cognitive Sciences.

[CR29] Ichikawa JJ, Jarvis BW (2009). Thought-experiment intuitions and truth in fiction. Philosophical Studies.

[CR30] Ichikawa JJ, Jarvis BW (2012). Rational imagination and modal knowledge. Noûs.

[CR31] Ichikawa JJ, Jarvis BW (2013). The rules of thought.

[CR32] Jenkins CS (2008). Modal knowledge, counterfactual knowledge and the role of experience. The Philosophical Quarterly.

[CR33] Kim M, Yuan Y (2015). No cross-cultural differences in Gettier case case intuition: A replication study of Weinberg et al. 2001. Episteme.

[CR34] Lane DJ, S R, Francioli S, Harris PL (2016). Children’s imagination and belief: Prone to flights of fancy or grounded in reality?. Cognition.

[CR35] Langland-Hassan P, Kind A, Kung P (2016). On choosing what to imagine. Knowledge through imaginaion.

[CR36] Levin J (2019). A case for the method of cases: Comments on Edouard Machery, philosophy within its proper bounds. Philosophy and Phenomenological Research.

[CR37] Machery E (2017). Philosophy within its proper bounds.

[CR38] Machery E, Stich S, Rose D, Alai M, Angelucci A, Berniūnas R (2017). The Gettier intuition from South America to Asia. Journal of Indian Council of Philosophical Research.

[CR39] Machery E, Stich S, Rose D, Chatterjee A, Karasawa K, Struchiner N, Sirker S, Usui N, Hashimoto T (2018). Gettier across cultures. Noûs.

[CR40] Machery E, Stich SP, Rose D, Chatterjee A, Karasawa K, Struchiner N, Sirker S, Usui N, Hashimoto T, Mizumoto M, Stich SP, McCready E (2018). Gettier was framed!. Epistemology for the rest of the world.

[CR41] Malmgren A-S (2011). Rationalism and the content of intuitive judgements. Mind.

[CR42] Nagel J (2012). Intuitions and experiments: A defense of the case method in epistemology. Philosophy and Phenomenological Research.

[CR43] Nagel J, San Juan V, Mar RA (2013). Lay denial of knowledge for justified true beliefs. Cognition.

[CR44] Nichols S, Nichols S (2006). Imaginative blocks and impossibility: An essay in modal psychology. The architecture of the imagination.

[CR45] Nichols S, Stich SP (2003). Mindreading: An integrated account of pretence, self-awareness, and understanding other minds.

[CR46] Nolan DP (1997). Impossible worlds: A modest approach. Notre Dame Journal of Formal Logic.

[CR47] NOS (2017a). De vijf meest gestelde vragen over fipronil, en de antwoorden. Online news article. Https://nos.nl/artikel/2187250-de-vijf-meest-gestelde-vragen-over-fipronil-en-de-antwoorden.html. (Accessed on 30 July 2018).

[CR48] NOS (2017b). Voedsel- en Warenautoriteit waarschuwt voor besmette eieren. Online new article. Https://nos.nl/artikel/2185885-voedsel-en-warenautoriteit-waarschuwt-voor-besmette-eieren.html. (Accessed on 30 July 2018).

[CR49] Pezzulo G (2011). Grounding procedural and declarative knowledge in sensorimotor anticipation. Mind and Language.

[CR50] Rafetseder E, Cristi-Vargas R, Perner J (2010). Counterfactual reasoning: Developing a sense of ‘nearest possible world’. Child Development.

[CR51] Roca-Royes S (2011). Modal knowledge and counterfactual knowledge. Logique et Analyse.

[CR52] Rysiew, P. (2017). Naturalism in epistemology. In E. N. Zalta (Ed.), *The Stanford encyclopedia of philosophy*. Metaphysics Research Lab, Stanford University.

[CR53] Sayadsayamdost H (2015). On normativity and epistemic intuitions: Failure of replication. Episteme.

[CR54] Shope R (1983). The analysis of knowing: A decade of research.

[CR55] Sosa E (2007). Experimental philosophy and philosophical intuition. Philosophical Studies.

[CR56] Sosa E, Borges R, De Almeida C, Klein PD (2017). The metaphysical Gettier problem and the X-Phi critique. Explaining knowledge: New essays on the Gettier problem.

[CR57] Starmans C, Friedman O (2012). The folk conception of knowledge. Cognition.

[CR58] Swain S, Alexander J, Weinberg JM (2008). The instability of philosophical intuitions: Running hot and cold on truetemp. Philosophy and Phenomenological Research.

[CR59] Turri J (2013). A conspicuous art: Putting Gettier to the test. Philosophers’ Imprint.

[CR60] Turri J, Hetherington S (2019). Experimental epistemology and “Gettier” cases. The Gettier problem.

[CR61] Turri J, Blouw P, Buckwalter W (2015). Knowledge and luck. Psychonomic Bulletin and Review.

[CR62] Vaidya, A. (2016). The Epistemology of Modality. In E. N. Zalta (Ed.) *The Stanford Encyclopedia of Philosophy*. CSLI Publications.

[CR63] Van Hoeck N, Watson PD, Barbey AK (2015). Cognitive neuroscience of human counterfactual reasoning. Frontiers in Human Neuroscience.

[CR64] Van Inwagen P (1998). Modal epistemology. Philosophical Studies.

[CR65] Vetter, B. (2017). Williamsonian modal epistemology, possibility-based. In J. Yli-Vakkuri, & M. McCullagh (Eds.) *Williamson on modality*, (pp. 314–343). Routledge.

[CR66] Walker CM, Gopnik A, Taylor M (2013). Causality and imagination. The Oxford handbook of the development of imagination.

[CR67] Weinberg JM (2007). How to challenge intuitions empirically without risking skepticism. Midwest Studies in Philosophy.

[CR68] Weinberg, J. M. (2017). Knowledge, noise, and curve-fitting. In R. Borges, C. de Almeida, & P. D. Klein (Eds.) *Explaining knowledge. New essays on the Gettier problem*, chap. 15, (pp. 253–272). Oxford: Oxford University Press.

[CR69] Weinberg JM, Nichols S, Stich S (2001). Normativity and epistemic intuitions. Philosophical topics.

[CR70] Williamson T (2007). The philosophy of philosophy.

[CR71] Williamson, T. (2014). What is naturalism? In M. C. Haug (Ed.) *Philosophical Methodology: The Armchair or the Laboratory?*, (pp. 29–31). Routledge.

[CR72] Williamson, T. (2016a). Knowing by imagining. In A. Kind, & P. Kung (Eds.) *Knowledge Through Imagination*, chap. 4, (pp. 113–123). Oxford University Press.

[CR73] Williamson, T. (2016b). Philosophical criticisms of experimental philosophy. In J. Sytsma, & W. Buckwalter (Eds.) *A Companion to Experimental Philosophy*, (pp. 22–36). Wiley Blackwell.

[CR74] Wright JC (2010). On intuitional stability: The clear, the strong, and the paradigmatic. Cognition.

[CR75] Wright JC (2013). Tracking instability in our philosophical judgments: Is it intuitive?. Philosophical Psychology.

